# Combining iron affinity-based fractionation with non-targeted LC-ESI-TOFMS for the study of iron-binding molecules in dissolved organic matter

**DOI:** 10.1093/mtomcs/mfac079

**Published:** 2022-10-10

**Authors:** Anna Rathgeb, Tim Causon, Regina Krachler, Stephan Hann

**Affiliations:** Department of Chemistry, Institute of Analytical Chemistry, University of Natural Resources and Life Sciences, Muthgasse 18, 1190 Vienna, Austria; Department of Chemistry, Institute of Analytical Chemistry, University of Natural Resources and Life Sciences, Muthgasse 18, 1190 Vienna, Austria; Institute of Inorganic Chemistry, University of Vienna, Althanstrasse 14, 1090 Vienna, Austria; Department of Chemistry, Institute of Analytical Chemistry, University of Natural Resources and Life Sciences, Muthgasse 18, 1190 Vienna, Austria

**Keywords:** dissolved organic matter, iron, immobilized-metal affinity chromatography, non-targeted analysis, high-resolution mass spectrometry

## Abstract

The low solubility of inorganic iron(III) in seawater leads to very limited availability of this important micronutrient for marine organisms. Estuarine or oceanic iron is almost entirely bound to organic ligands of mainly unknown chemical structure. In this context, riverine input of iron rich, land-derived dissolved organic matter (DOM) can play an important role in coastal areas and investigation of potential Fe-ligands in DOM is of high interest. Previous studies have suggested that iron is predominantly bound to the high molecular weight fraction of DOM, but distributed over the entire size range. Logically, structural elucidation needs to start from the smallest building blocks. A model study targeting low molecular weight iron-binding constituents in Suwannee River natural organic matter (NOM) using Fe-loaded Chelex or silica for immobilized-metal affinity (IMAC)-based fractionation was undertaken. The binding strengths of different compounds could be qualitatively assessed using a differential analysis workflow. IMAC-fractionated samples were acidified and analyzed via liquid chromatography high resolution mass spectrometry (LC-HRMS) and molecular formulas were assigned using state of the art software. A total of 144 Fe-binding constituents in Suwannee River NOM were found to be of interest with the largest number observed to interact with Chelex at pH 4 (55%), and the smallest with silica at neutral pH (24%). Most binding constituents were found in the lignin- and tannin-type region of the van Krevelen plot. Results from this study support the hypothesis that very low molecular weight constituents (below 300 Da) can play a role in the iron binding mechanism of DOM and demonstrate that the employed analytical workflow is suitable for their detection.

## Graphical Abstract

**Graphical Abstract:**
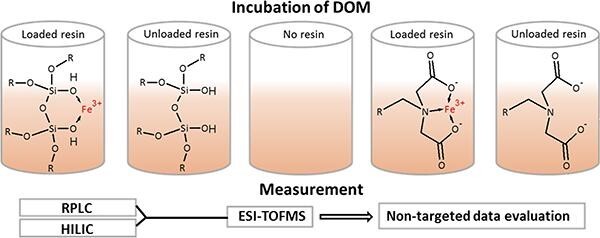
Iron affinity chromatography supported non-targeted analysis of iron-binding molecules in natural waters.

## Abbreviations

DOM: dissolved organic matter
IMAC: immobilized-metal affinity chromatography
MS: mass spectrometry
NOM: natural organic matter
RT: retention time
s.w.: seawater
SR NOM: Suwannee River NOM

## Introduction

Some decades ago, iron deficiency was identified as a growth limiting factor in several oceanic regions.^1^ Iron is an essential micronutrient and the low solubility of inorganic Fe(III) leads to a limited supply for marine organisms.^2^ Dissolved iron in the ocean is almost entirely bound to organic ligands which, apart from a few well-characterized siderophores, are largely unknown or poorly characterized.^3^^,^^4^ Similarly, the importance of land-derived organic matter for iron transport in the mixing zone is widely accepted,^5–7^ but the chemical nature of Fe-binding ligands capable of transporting iron to coastal waters remains poorly understood.

While the results of several analytical studies utilizing size-exclusion chromatography or field-flow fractionation have suggested that iron is predominantly bound to the high molecular weight fraction of DOM,^8–10^ a closer look at the small molecules present in DOM is worthwhile for several reasons. Firstly, a significant proportion (up to 35% w/w) of Fe is reported to be in the fraction below 1 kDa,^9^^,^^10^ while there are also indications that high molecular weight DOM constituents in fact partly consist of associated small molecules held together by hydrophobic interactions, pi-pi stacking, hydrogen bonding, or as coordination compounds.^11–14^ Secondly, structure elucidation of low molecular weight, free ligands can be achieved more easily than for larger constituents when using mass spectrometry. Thus, one goal of ongoing studies in this area is to build up a solid basis of well-described DOM constituents and subsequently move on to the investigation of larger clusters.

The direct analysis of DOM-Fe associates in iron-rich samples using molecular mass spectrometry is an attractive analytical approach, but several drawbacks are apparent. Firstly, the stability of iron complexes is limited by pH and by the electrospray process itself, which may introduce gas phase dissociation and recombination reactions leading to artifacts and misinterpretation. Moreover, it can be assumed that in many cases more than one ligand is bound to a central iron atom as a coordination compound, which can (partly) dissociate in the ESI source during analysis. As a further mass spectrometry (MS)-related difficulty, transition metal complexes are often charged, and, if the net charge of the coordination compound is unknown (e.g. due to an unknown number of negatively charged ligands like organic acids) or not an integer, correct molecular formula assignments are not possible.^15^ Additionally, chromatographic separation of labile or charged compounds is not particularly rugged, especially in the case of iron, which is prone to low solubility at increasing pH while its coordination compounds are often unstable at low pH. In general, charged complexes are not retained by reversed-phase liquid chromatography (RPLC) which means that less selective methods such as ion chromatography need to be applied. On the other hand, larger polydentate complexes are more stable and are amenable to LC-MS analysis. A recent study has demonstrated the capabilities of liquid chromatography high resolution mass spectrometry (LC-HRMS) for characterization of DOM-Fe associates.^16^

Thus, in order to focus selectively on iron-binding DOM constituents while avoiding the issues described above, a method utilizing immobilized metal-affinity (IMAC) principles is appealing. The most frequently used material to perform IMAC is Chelex, a cross-linked beaded polystyrene resin carrying an iminodiacetatyl functional group.^17^ Previously, Burba *et al*.^18^ investigated DOM on iron-loaded Chelex performing pH gradient elution from 4 to 12. With increasing pH, DOM constituents are replaced by OH^−^ ions, but the authors found that approximately 10–15% of the sample could not be eluted with this approach. A large majority of existing publications involving IMAC and DOM deal with Cu-loaded resins.^19–23^ Available organic ligands have a major effect on the bioavailability and toxicity of Cu, which binds strongly to the Chelex resin. Within these studies, elution strategies using pH gradients or competing ligands were developed and the potential of DOM to interact with transition metals was demonstrated. However, despite the emerging understanding of DOM-mediated iron transport in the general literature, only few studies targeting interactions with Fe-loaded resins have been conducted to date.

Apart from Chelex, other approaches to fractionate DOM exploiting iron affinity have also been investigated. For example, an interesting work was conducted by Polubesova *et al*.^24^ in which a clay sorbent was loaded with iron and incubated with DOM. The supernatant was analyzed by Fourier transform infrared spectroscopy (FT-IR) and compared to the original solution. It was found that aromatic-, carbonyl-, and amine-specific vibrations were diminished, whereas polysaccharides did not seem to be adsorbed by the resin. Other studies have investigated how the affinity of DOM for iron can be exploited regarding wastewater treatment. Within this context, the DOM fraction not coagulating with iron salts was analyzed via electrospray ionization Fourier transform ion cyclotron resonance mass spectrometry (ESI-FT-ICRMS). The results were in good qualitative agreement with the study conducted by Polubesova *et al.*^24^ and showed that oxygen-rich, less saturated, and nitrogen-rich compounds were preferably removed.^25^ Moreover, it was confirmed via gas chromatographymass spectrometry (GC-MS) analysis that polysaccharides remain unaffected and the same was observed for proteins.^26^ Also focusing on DOM removal, Moriguchi *et al*.^27^ developed a procedure to load silica with iron and characterized the resultant material concerning pH-dependent surface charge, which was determined to be slightly positive from 2 to 7. In this case, the geometry of the silica-bound iron is not fixed, meaning that nature of the coordination of iron is likely to be influenced by pH and ionic strength. The authors found this material to be a very promising IMAC adsorbent binding up to 86% of fulvic and 97% of humic acid constituents at mg/g ratios.

In the case of Chelex, iron is chelated by the amine and two carboxyl groups of iminodiacetic acid in one plane.^23^ Three binding positions remain available to form a coordination compound with octahedral geometry as typical for iron. The affinity of compounds toward Chelex-immoblized Fe may differ from the behavior towards free, ionic iron. For this reason, we considered it as crucial to test more than one type of IMAC material in order to focus on compounds with unambiguous iron affinity, and also to minimize the influence of any resin-dependent artifacts. IMAC is essentially a fractionation approach providing several broad zones at best, but if ESI-TOFMS analysis of complex mixtures is to be performed, combination with an analytical-scale chemical separation is extremely useful for several reasons. Most prominently, space-to-charge effects, artifact formation in the ion source, and the degree of complexity of the MS spectra are all reduced, while isotopologue and adduct assignments by software-based alignment of single ion features can be realized.

In a previous study, we focused on the LC-MS-based characterization of DOM from various origins without direct reference to iron affinity.^28^ Thus, in the framework of the present study, we aim to close that gap and report an investigation of ligands isolated via fractionation based on iron affinity. It is important to note that the presented workflow is an indirect approach. Hence, the applied LC-HRMS method is not targeting the detection of iron complexes; only free ligands are separated and detected. To this end, a model study employing incubation experiments of Suwannee River natural organic matter (SR NOM) with Fe-loaded Chelex and silica resins in combination with non-targeted high-resolution LC-ESI-TOFMS analysis assessed using a differential metabolomics-type workflow was undertaken.^29–32^

## Materials and methods

### Chemicals

The following substances were obtained from Sigma-Aldrich or Fluka (Vienna, Austria): Chelex 100 sodium form 50–100 mesh, naringenin, 2,5-dihydroxybenzoic acid, magnesium chloride hexahydrate (≥99.0%), calcium chloride dihydrate, sodium sulfate (≥99.5%), potassium chloride (≥99.5%), sodium hydrogen carbonate (≥99.5%), potassium bromide (99%), boric acid (≥99.5%), strontium chloride hexahydrate (99.995%), sodium fluoride (99.99%), ammonium hydroxide (28% v/v, purified by double distillation), iron(III) chloride hexahydrate (≥99.0%), ammonium hydrogen carbonate (≥99.5%), ammonium formate (≥99.0%), silica gel (high-purity grade, pore size 60 Å, 70–230 mesh, 63–200 μm). Sodium chloride (≥99.5%), formic acid (98-100%), nitric acid (65%, ultrapure), and indium ICP standard were purchased from Merck KGaA (Darmstadt, Germany). LC-MS grade methanol was purchased from Fisher Scientific (Vienna, Austria). The Fe(III) single element standard in 2% v/v nitric acid used for the external five-point calibration was obtained from PerkinElmer.

### Preparation of Fe-loaded Chelex and incubation with DOM

Three conditions, pH 4 with 10 mM NH_4_COOH buffer, neutral pH with 10 mM NH_4_HCO_3_ buffer (pH 7 for silica, pH 8 for Chelex), and 50% artificial seawater (prepared based on a recipe proposed by Kester *et al*.^33^) were tested. These conditions were chosen to determine not only whether a component interacts with Fe-loaded material, but also to draw some qualitative conclusions about the binding strength. The experiments using Chelex and silica resins were conducted in the same way to allow direct comparisons to be made. Detailed information is provided in the following sections.

Chelex 100 sodium form 50–100 mesh was loaded by adapting the procedures provided by the manufacturer^17^ and those reported by Burba *et al*.^18^ A ratio of 1 mg Fe to 1 mg DOM was sought as proposed by Burba *et al*.^18^ According to the manufacturer, 90 mg Chelex are needed to bind 1 mg Fe, so an excess of 30% above the manufacturer recommendations was used to ensure that there was free capacity to take up free iron introduced by the SR NOM samples, or arising from contamination of final sample solutions. Iron(III) chloride hexahydrate was weighed in and dissolved in a few drops of concentrated formic acid and this solution was subsequently diluted with ultra-pure water to an approximate concentration of 40 mM and adjusted to pH 3.2 with 28% v/v ammonium hydroxide solution after the addition of Chelex. The suspension was stirred for 2 h, during which the solution decolorized and the Chelex phase turned deep red. Aliquots of the original Fe-solutions and the Chelex supernatants were taken, diluted 1:100 000, and directly infused into an iCapQ ICP-QMS mass spectrometer operated in KED (kinetic energy discrimination) mode. The Fe-signal intensity of the incubated solutions was <1% (total counts) compared to the original solutions and therefore the loading was considered as complete. Chelex, regardless if Fe-loaded or untreated, was washed with 3% v/v HCOOH and then with ultra-pure water (10 ml for 1 g, three times each) before each experiment. Three independent batches were prepared to determine precision under repeatability conditions. Solvent was removed using a pipette before Chelex was weighed into tubes and the sample was added. However, as the remaining resin was still moist, some dilution of the sample was unavoidable. As the present study aimed at relative quantification via fold-change assessments, a portion of Chelex was dried at 60°C until constant weight was reached (24 h) and the concentration of the sample stock solution was adjusted accordingly (the dried Chelex was discharged).

For the incubation experiment, a 12.5 g/l stock of SR NOM was prepared by dissolving the powder in 2% v/v NH_4_OH, which was then diluted 1:10 with buffer at pH 4 or pH 8 or with 50% artificial seawater at pH 8. The final volume of the incubation solutions was 2.5 ml with 300 mg Chelex and approximately 1 g/l DOM. Overall 18 incubation samples were prepared; this included three replicates of the three conditions incubated with treated or untreated Chelex. Moreover, 18 incubation blanks (meaning that the experiment was performed in exactly the same way, but adding buffer/seawater instead of DOM) were prepared.

Solutions were continuously stirred and incubated for 10 min to allow the system to reach equilibrium and then the pH was readjusted carefully with diluted NH_4_OH or HCOOH when necessary. Once the pH remained constant (i.e. ± 0.2), a further 50 min of stirring was applied. Samples were subsequently syringe filtered over 0.45 μm to remove the resin, acidified to a final concentration of 3% v/v HCOOH to (i) dissociate possibly existing traces of Fe-complexes and (ii) to protonate weak organic acids and increase retention on the RPLC column, again syringe-filtered over 0.45 μm to remove potential precipitate upon acidification, spiked with the internal reference compounds naringenin and 2,5-dihydroxybenzoic acid (each 2 μM final concentration), and transferred into high-performance liquid chromatography (HPLC) vials.

Approximately 200 μl of each sample (not including the blanks) were mixed to yield a pooled sample, which was subsequently aliquoted. The samples were stored at −40°C until measurement. On the day of measurement, fresh samples were prepared in the same manner, except that no material was added prior to the incubation step and the sample was stirred for 50 min. One fresh sample for each condition (pH 4, neutral, and 50% seawater) was prepared. Aliquots of these sample solutions were then added to the pooled samples after thawing. Thus, effects based on freezing and thawing or unspecific interaction with the material could be accessed and compounds showing such behavior were later removed from the dataset.

### Preparation of Fe-loaded silica and incubation with DOM

The preparation of Fe-loaded silica was adapted from Moriguchi  *et al*.^27^ Silica was slurried in water and FeCl_3_ solution was added. The w/w ratio of Fe: silica was 1:10 mg, and the iron concentration was approximately 200 mM. The pH was adjusted to 3.5 with 14% v/v NH_4_OH, and the mixture was stirred at ∼80 °C for 24 h. The FeCl_3_ solution was filtered off, and the silica was washed with 3% v/v HCOOH to remove excess iron and subsequently rinsed with water. The obtained dark red product was dried over 24 h at 170 °C. As a ratio of 1 mg Fe:1 mg DOM was desired, the bound iron needed to be quantified. Therefore, 1 mg of Fe-loaded and untreated silica was dissolved in 9 ml of 2% v/v NH_4_OH, and 1 ml concentrated HCOOH was added to re-dissolve the iron (under acidic conditions, no binding of Fe to the material occurs). The solutions were diluted 1:20 in 2% v/v nitric acid and spiked with 1 μg/l indium solution for internal standardization. An external calibration in the range 0–100 μg/l Fe was prepared. Approximately  1 mg loaded silica contained 33 μg, whereas the unloaded silica contained 0.45 μg Fe. This indicates that about 30 mg of silica are needed to bind 1 mg Fe. Thus, silica was weighed in based on the desired Fe: DOM ratio (1 mg Fe:1 mg DOM) of the loaded material and the same amount of untreated material was used. The workflow used was identical to 2.2., except that pH 6.8 instead of 8 was used for the incubation in NH_4_HCO_3_ buffer and seawater to minimize dissolution of the silica gel and electrostatic repulsion of DOM by negatively charged silanol groups on the surface.

### Analytical measurements

ICP-MS experiments were conducted on an iCapQ in KED (kinetic energy discrimination) mode (Thermo Fisher, Bremen, Germany) or an Elan DRC II with methane reaction cell (PerkinElmer SCIEX, Ontario, Canada). The measurement accuracy, expressed as the precision under repeatability conditions of measurement and trueness (in terms of relative bias) of the ICP-MS measurements, was approximately 5% and 10%, respectively.

For pH measurements, a PHM92 pH-Meter (Radiometer, Copenhagen, Denmark) with a micro electrode (inLab Micro, Mettler, Toledo, USA) was used.

A dual-column LC-MS method adapted from Klavins *et al*.^29^ was run on an HPLC System with two gradient pumps and a 10-port valve (Agilent Infinity II, Agilent Technologies, Santa Clara, USA). An Atlantis T3 C18 (3 μm particle size, 150 × 2.1 mm) and an Atlantis T3 guard column (3 μm, 20 × 2.1 mm) from Waters (Milford, USA) were used for RPLC separation, while a Nucleodur column (1.8 μm, 100 × 2 mm) from Macherey-Nagel (Düren, Germany) was employed in parallel for hydrophilic interaction liquid chromatography (HILIC). The sample was injected on the RPLC and the HILIC column simultaneously (5 μl each), and the column effluents were combined before entering the ESI interface of the TOFMS. The linear RPLC gradient was run with (A) 0.1% v/v HCOOH and (B) MeOH and switched back to starting conditions after 14.10 min. The HILIC gradient was run with (A) 10 mM ammonium formate pH 3.25 and (B) acetonitrile and switched back after 6.10 min. Thus, all peaks eluting after 7 min can be assigned to the RP column.

Negative mode mass detection was performed using an Agilent 6230 TOFMS (6230B Time of Flight (TOF) LC/MS, Agilent) equipped with a Dual Jet Stream ESI source. The following source parameters were set: 120 °C drying gas temperature with 10 l/min flow rate, 45 psig nebulizer pressure, 350 °C sheath gas temperature, and 12 l/min flow rate. The capillary, fragmentor, and skimmer voltages were set to 3500, 120, and 60 V, respectively. The detector was operated in the 2 GHz extended dynamic range mode, with two full spectra per second recorded. Profile MS data were acquired within a mass range of 100–1600 *m/z* employing two reference masses for mass drift correction.

After every sample, a 3% v/v HCOOH blank was injected to avoid carry over and to keep the system clean over the analysis time of around 30 h per batch. Three technical replicates of the fresh samples were measured, while each of the three incubated sample replicates was measured once.

### Data processing and evaluation

Feature detection and alignment were performed with Agilent MassHunter Profinder B.08.00 using batch recursive feature extraction. This process entails an initial molecular feature finding (step 1), followed by creation of a target list for recursive extraction of all samples based on the molecular feature list generated (step 2). A detailed description of this workflow is given in a previous publication.^28^

In step 1, a chromatographic retention time window of 0.9 to 16.0 min was defined. Detected (single) ions were filtered *via* height (≥ 30 counts). For grouping of isotopologues and adducts, a peak spacing tolerance of ± 0.0025 *m/z* or ±7 ppm was set, formate adducts were allowed, and the software ‘common organic isotope’ model was used. At least two signal features (ions) were required for a signal to be considered as a molecular feature. Background spectra from the baseline signal close to the chromatographic peak were subtracted. In step 2, a retention time window of ± 0.3 min and a mass window of ±20 ppm was allowed.

All components with distinct peaks in any blank were removed from the datasets. Finally, the data were exported to MassHunter Qualitative Analysis B.07.00. The elements C, H, O, N, S, and P were considered for molecular formula annotation The score calculated by the Agilent Mass Hunter software for the generated formulas based on the probabilities for the mass, isotope abundance, and spacing. The scoring was weighted according to mass accuracy (100), isotopologue spacing (50), and isotopologue abundance (50). An overall score of at least 70 on a scale of 0 to 100 had to be achieved; otherwise, the molecular formula was rejected. Up to 5 ppm mass error were allowed. Formula results were checked via ChemSpider to determine whether the obtained molecular formulas were chemically reasonable for the given samples. Moreover, extracted ion chromatograms of all hits were extracted manually in the software to ensure that no interferences in the profile MS spectra were present. With these steps wrong assignment of co-eluting compounds can be significantly reduced. Corresponding to Schymanski *et al*.^34^ this procedure reveals confidence level 4 ( = unequivocal molecular formula).

The obtained molecular formula lists from the Chelex and the silica batch were combined to create an in-house database using Agilent PCDL Manager B.07.00. Subsequently, a targeted extraction using Profinder software was performed. For software-based extraction, a mass window of 15 ppm and a retention time window of 0.3 min were employed. Reliable compounds had to be present and of high quality in both datasets to be further considered in the workflow. The fresh samples, the samples incubated with untreated material and those incubated with Fe-loaded Chelex and silica resin, respectively, were compared for each condition (pH 4, 7/8 and seawater for both materials). Finally, the annotated molecular formulas were divided into five groups. The group assignment process is expressed via the variable ‘a_n_(f)’ representing chromatographic peak area of a compound *n* detected in the fresh untreated sample (see Table 1).

- **Group 1: Uninfluenced.** No significant changes in chromatographic peak area. Considered to have no interaction with the resin.
- **Group 2: Unspecific interaction.** Found to only be present in fresh sample or the chromatographic area was reduced by at least a factor of 2 in the sample incubated with untreated material compared to the sample incubated with Fe-loaded material. Indicates non-specific interaction with the material or loss caused by freeze-thawing. Unspecific interaction with the untreated material may also include possibly existing Fe-complexes in the case of the samples with pH 7 and pH 8 as well as the seawater samples. However, this is not the case at pH 4, where Fe-complexes are not stable. Hence, ligands that were initially present in complexed form in seawater or at higher pH will not be overlooked by this approach.
- **Group 3a: Strong Fe-mediated interaction (not detected after incubation).** Component was no longer detected after incubation with Fe-loaded material, but was found to be present in the sample incubated with untreated material and in the fresh sample.
- **Group 3b: Moderate Fe-mediated interaction.** Chromatographic peak area in the sample incubated with unloaded material was at least double the area of the sample incubated with Fe-loaded material. Either compound does not interact with Fe as strongly as in group 3a or the compounds abundance is high and thus detection is still possible, even if a high proportion is removed.
- **Group 4: Absent.** Component was not detected in this sample group.

**Table 1.** Differential approach for classification of compounds according to incubation experiment results with the two resins. Compounds were assigned to a group according to the changes in chromatographic peak area with reference to the corresponding peak area obtained for a fresh, untreated sample, a_n_(f) = chromatographic peak area of compound n in the fresh, untreated sample, n.d. = not detected

	Fresh sample	Unloaded resin	Fe-loaded resin
Compound group 1: Uninfluenced	a_n_(f)	= a_n_(f)	= a_n_(f)
Compound group 2: Removed due to unspecific interaction with unloaded solid phase	a_n_(f)	≤ a_n_(f)/2	= a_n_(f), or matches unloaded resin
Compound group 3a: Selectively removed by Fe-loaded resin	a_n_(f)	= a_n_(f)	n.d.
Compound group 3b: Fe-mediated fold change	a_n_(f)	= a_n_(f)	≤ a_n_(f)/2
Compound group 4: Absent	n.d.	n.d.	n.d.

## Results and discussion

In the following sections, the results of the incubation experiments on Fe-loaded Chelex and silica under three conditions including pH 4, pH 8 (Chelex) or pH 7 (silica) and 50% v/v artificial seawater at pH 8 or 7 are presented and discussed. These different conditions were tested because strongly binding ligands can be discriminated from weakly binding ones via pH,^18^ while the effect of a high salt matrix allows to study inferences for ligands able to bind to iron in coastal areas.

### Summary of differential analysis results

In our study, the accurate assessment of the amounts of iron loaded in the different experiments were considered crucial, especially for silica, for which only limited data are available so far. For both materials, pH is an important parameter and pH 3.5 was found to deliver the highest iron-binding capacity. For silica, a maximum iron loading of 3.3% w/w was reached. For Chelex, the capacity was assumed to be 1.1% w/w according to the manufacturer. For both resins, the corresponding molecular formulas of the quality control compounds (naringenin and benzoic acid) were assigned correctly and the mass errors did not exceed 1.3 ppm throughout the study.

**Fig. 1:**
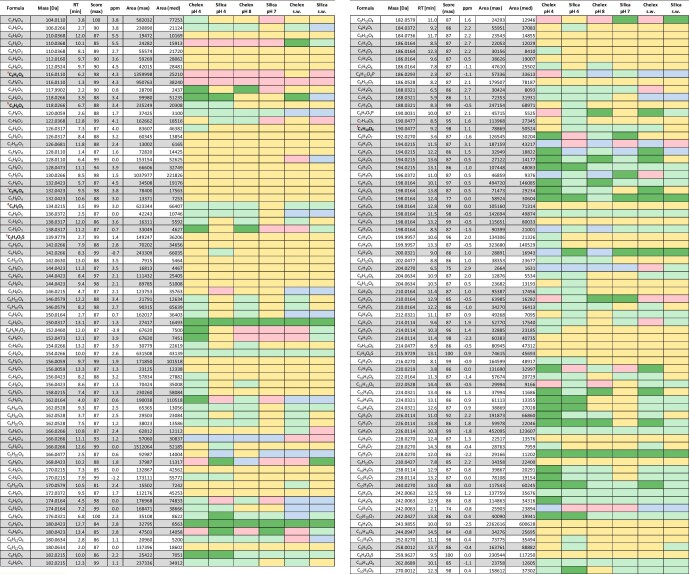
Color coded list containing all assigned compounds showing the calculated molecular formula, exact monoisotopic mass, retention time, software quality score, mass error in ppm, and the maximum and median of the chromatographic peak areas. Color coding is according to the approach described in Table 1. The red letters a–f are indicating the compounds listed in Table 2.

In Fig. 1 all compounds detected via the Fe-affinity-NTA workflow are listed and categorized according to the scheme explained above. Many of the detected small molecules show apparent, selective interaction with iron on both resins investigated (i.e. groups 3a and 3b). Within this list, it is of note that several molecular formulas are annotated more than once and are displayed as gray and white blocks. These entries represent structural isomers and often show very different behavior towards the resins highlighting the importance of using chromatography in accurate MS workflows for NOM-related studies.

Using the dual-column LC-TOFMS workflow, every component in a given sample can be theoretically detected twice; once from the RP and once from the HILIC column. Practically, this was only observed in a small number of cases as substances strongly retained on one column often elute in the void of the other due to the orthogonal retention mechanisms of the two columns. Additionally, peaks eluting from the HILIC column are characteristically broader, and less symmetric than RPLC, which supports identity confirmation in such cases. Once confirmation of the same molecular formula eluting from both columns was made, the group assignment was compared and, if it was found to be identical, only the entry with stronger chromatographic retention was maintained, while the second entry was removed from the dataset. This situation only applied to a total of five compounds. There are several molecular formulas listed more than once in the final list which indicates the presence of structural isomers, which could be successfully resolved due to the chromatographic separation.

It was also observed that the high salt concentration in the 50% v/v artificial seawater samples led to ion suppression and, in addition, hampered chromatographic separation in some cases. Nevertheless, a substantial fraction remains chromatographically accessible and shows strong interaction with the Fe-loaded resin despite the presence of the competing ligands in the matrix. The compounds exhibiting Fe-interaction on both resins under all tested conditions are the most promising, and further structural elucidation is of high interest.

### Behavior of previously identified compounds in the Fe-affinity-NTA workflow

In an earlier study, several low molecular weight DOM compounds have been detected and their identity has been confirmed via spiking experiments using authentic standards in combination with the identical LC-ESI-TOFMS method.^28^ Within the present work, these compounds were again successfully detected and annotated by the software algorithms and can therefore serve as quality control parameters regarding the reproducibility of the workflow (Table 2).

**Table 2.** Substances identified in a previous study^28^ and confirmed in the present work

	Substance	Retention time (min)	Separation
a	Maleic acid	6.2	HILIC
b	Succinic acid	6.7	RPLC
c	Glutaric acid	9.5	RPLC
d	Malic acid	3.5	HILIC
e	Sulfoacetic acid	2.7	HILIC
f	3-Carboxyadipic acid	9.2	RPLC

Of these previously identified compounds, succinic acid (p*K*_a1_ = 4.2, p*K*_a2_ = 5.6), malic acid (p*K*_a1_ = 3.4, p*K*_a2_ = 5.2), and 3-carboxyadipic acid were found to exhibit some interactions with Fe-loaded material. Succinic acid only exhibited strong interactions with loaded resins at pH 4, while malic acid and 3-carboxyadipic acid were also observed to interact strongly at higher pH values, indicating that they are relatively stronger ligands. In terms of structure, succinic acid and malic acid share the same carbon backbone, but malic acid has an additional OH-group thus providing one additional site for Fe interaction. Thus, this result is in good qualitative agreement with the expectation that malic acid is a stronger ligand. This also confirms that the behavior toward the resins at higher pH values is primarily based on Fe-affinity and not due to electrostatic repulsion as the opposite elution behavior would be expected.

### Chemical properties of compounds with Fe-affinity

**Fig. 2:**
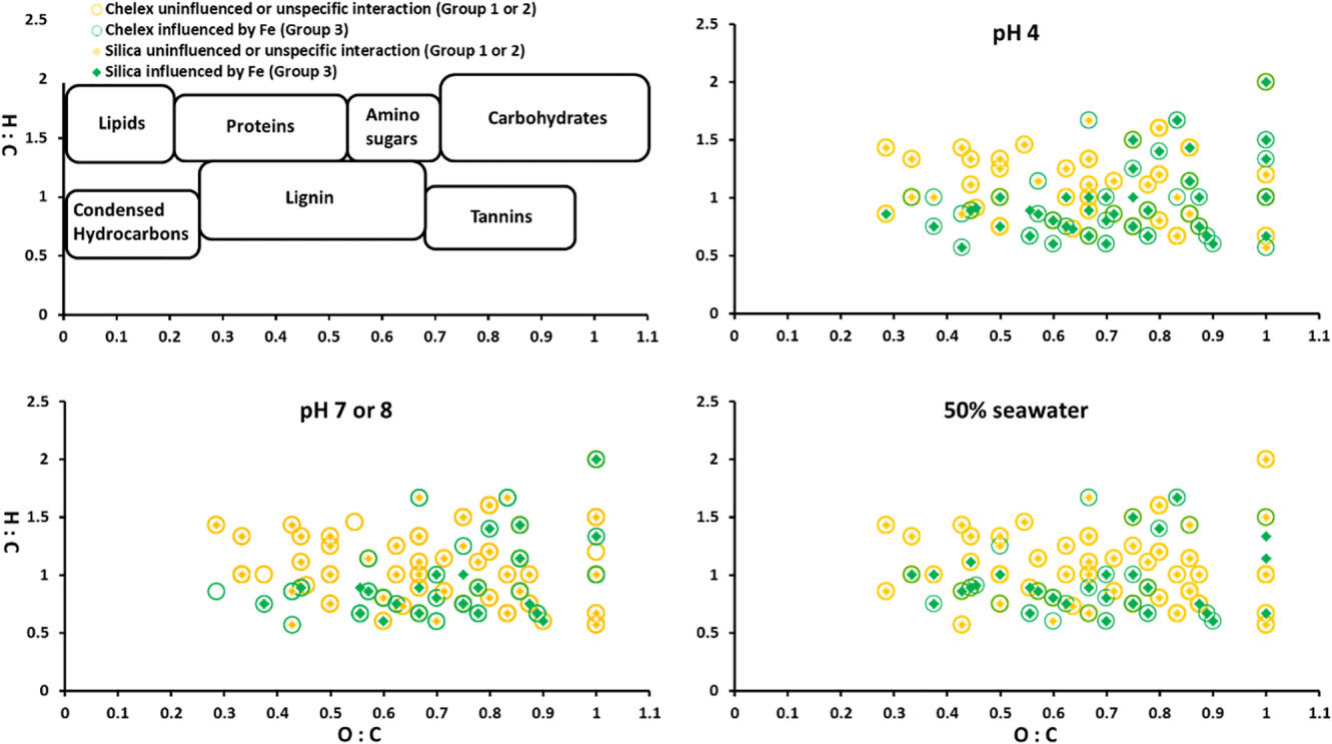
Van Krevelen plots for annotated compounds at all experimental conditions on both resins. Each point corresponds to a single compound with a retention time. If a molecular formula was listed several times in Fig. 1, then the one showing the strongest Fe-interaction is depicted.

The total number of compounds and the percentage associated to groups 1–3 are given in Table 3. Using the annotated molecular formulae of these compounds, the percentage of oxygen-rich iron-interacting compounds was calculated for each tested condition. Consequently, it becomes clear that the number of Fe-interacting compounds is increasing with the number of oxygen atoms in the formulas (Table 3 and Fig. 2). Furthermore, Van Krevelen plots based on stoichiometric ratios (H : C versus O : C) are a widely used approach for data illustration in DOM research, for example to compare samples of different origins^28^ or in connection with incubation experiments^25^ such as in the present study. In these plots, specific ratios correspond to certain substance classes or their degradation products. It is well known that surface river water DOM mainly contains lignin- and tannin-type compounds (Fig. 2)^35^^,^^36^ and also carboxyl-rich alicyclic molecules (CRAM)-type molecules.^37^ It is important to mention that classification in distinct molecular categories is limited regarding accuracy, as DOM is not fully characterized at a molecular level. An alternative classification has been proposed by Koch and Dittmar.^38^

**Table 3.** Summary of the group assignment results following software-based extraction of grouped molecular features. The proportion of Fe-interacting compounds decreases with rising pH and more compounds interact with the loaded Chelex compared to silica under all conditions

	Chelex pH 4	Silica pH 4	Chelex pH 8	Silica pH 7	Chelex seawater	Silica seawater
Total number of compounds detected	137	138	136	139	115	129
Group 1: Uninfluenced	38	43	53	65	58	62
Group 2: Removed due to unspecific interaction with unloaded solid phase	7	7	7	10	14	5
Group 3a: Removed by Fe	22	13	9	7	13	5
Group 3b: Fe-mediated fold change	33	37	32	17	16	27
% Group 3	55	50	42	24	29	33
% of 3 O:C ≥ 0.8	47	35	40	32	36	26

Each data point shown in the provided van Krevelen diagrams corresponds to a single compound with a given retention time. If a molecular formula is listed several times in Fig. 1, then only the one showing the strongest Fe-interaction is depicted in Fig. 2.

Fig. 2 indicates that the highest percentage of compounds interact with the Fe-loaded materials at pH 4 due to (i) dissociation of Fe-complexes and (ii) reduced competition with OH^−^ ligands, which is in good agreement with previous observations made by Burba et al.^18^ Most compounds in this condition are oxygen-rich and have a low degree of saturation, which is also concordant with previous findings.^19^^,^^25^

### Influence of pH and ionic strength on iron affinity

At higher pH values, the percentage of compounds observed in group 3 drops from 55% to 42% for Chelex and from 50% to 24% for silica. In 50% artificial seawater (high pH and high ionic strength), 29% of compounds detected interacted with the Fe-loaded Chelex and 33% with the loaded silica. Also confirmed was that the average O : C ratio of compounds decreased for both materials with increasing pH and ionic strength. Ideally, this result should only be a reflection of the Fe-DOM binding strength, but other effects cannot be comprehensively ruled out. For example, Moriguchi *et al*.^27^ reported a slightly positive, but decreasing zeta potential of loaded silica from pH 2 to 7. However, this still this does not guarantee the negligibility of electrostatic repulsion between multiply-charged anionic ligands (more likely to be found at high O : C ratios) and the resin. On the other hand, the number of components interacting with the unloaded materials was found to remain more or less constant or even rose with pH. Burba *et al*.^18^ also observed only a weak pH dependence of the interaction of DOM with unloaded Chelex. Moreover, it is worth noting that Fe-loaded silica has lower capacity at pH 4 and 7 in comparison to Chelex. At pH 4, the zeta potential of the loaded silica is positive, but the percentage of oxygen-rich compounds interacting is still much lower than on Chelex (35% versus 47%).

Increasing ionic strength was found to hamper the DOM-Fe interaction on Chelex substantially (29% versus 42% at pH 8) and promoted unspecific interactions. However, these conditions had the opposite effect on silica (33% versus 24% at pH 7). We propose that this has to do with the different bonding nature of the Fe-resin interactions. Moriguchi *et al*.^27^ previously proposed a combination of 1- and 2-fold coordination of iron via SiO^−^, meaning that more free binding locations would be available (in comparison to Chelex). Artificial seawater was the only condition where iron-loaded silica proved to be a more efficient resin than Chelex suggesting that experiments with heavy matrices should be preferably conducted with Fe-loaded silica. The present approach is suited to directly investigate seawater or estuarine samples, and the risk of losing compounds via typically employed desalting and isolation can be avoided. This is especially relevant when highly polar and low molecular weight DOM is targeted because the recovery for this fraction is often poor.^39^

**Fig. 3:**
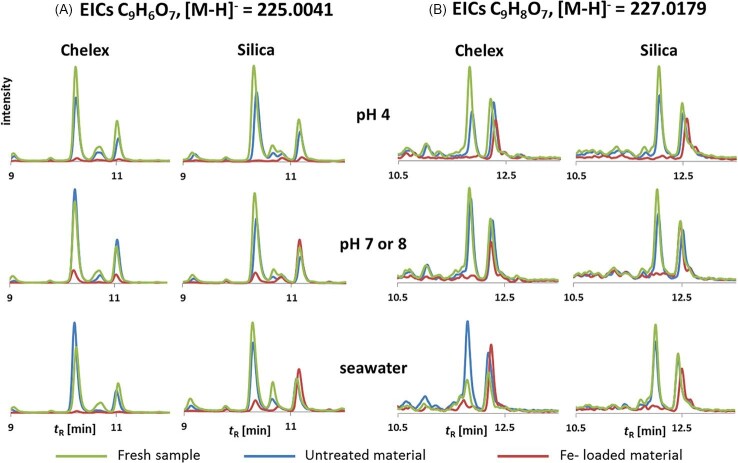
Two examples of chromatographic separation of isobaric/isomeric compounds demonstrated to be necessary for correct discrimination of varying Fe-interaction behavior. A mass extraction window of 20 ppm for the monoisotopic ion was used.

Thus, for compounds found to interact with the resins at pH 4 and neutral conditions, but not in artificial seawater, it is not feasible to conclude that the compound would bind to iron in the river and later dissociate in the estuarine mixing zone. Conversely, compounds observed to interact with the loaded resins in artificial seawater despite possible repulsion from the resin and competition of the matrix for the free binding sites of iron can be assumed to have a very strong affinity towards Fe. Overall, many compounds belonging to group 3 for both materials under all tested conditions can therefore be considered as being iron binding and of significance for transport in natural waters. Two examples of such compounds are shown in Fig. 3.

In Fig. 1 of the clear advantages of using chromatographic separation in combination with high resolution MS is demonstrated. In both examples 3A and 3B, the measured accurate mass corresponds to more than one compound present in the sample. For example, the first chromatographic peak corresponding to the formula C_9_H_8_O_7_ is always assigned to group 3a, while the second peak is assigned to group 1, meaning that no significant interaction with iron was observed. These observations illustrate that coordination chemistry depends strongly upon functional groups present and molecular geometry and underpin the critical importance of high chromatographic resolution in such studies. Functional groups are, to a certain degree, assessable using FT-IR,^24^ but geometry can only be defined after unequivocal identification of the relevant compound. 

## Concluding remarks

In the present work, we could show that DOM-derived small molecules play a role regarding the interaction with iron in natural waters. The incubation experiments performed with two different iron-loaded resins allowed successful fractionation of low molecular weight components of DOM characterized by strong affinity to iron. Results obtained from a differential analysis approach following RPLC and HILIC separations in combination with ESI-TOFMS demonstrated that the developed workflow is well suited for detecting and characterizing the analytes of interest and displays higher selectivity than direct infusion, particularly when isomeric constituents are in play. The hypothesis that the low molecular weight size fraction plays a role in DOM-mediated iron mobilization either as individual ligands or as part of larger building blocks is supported by the results from this study. In particular, oxygen-rich and unsaturated compounds in the lignin- and tannin- region were retained on the loaded resins, while results indicate that lignin-type ligands bind more strongly than other detected classes. For future work, high abundance compounds showing strong and selective interaction with iron under all experimental conditions with both resins should be further investigated using high-resolution mass spectrometry with fragmentation with the goal to elucidate their structures as van Krevelen plots can only provide first hints in this undertaking.

## Data Availability

The data underlying this article will be shared on reasonable request to the corresponding author.
